# The Chromatin Remodeling Factor *BrCHR39* Targets DNA Methylation to Positively Regulate Apical Dominance in *Brassica rapa*

**DOI:** 10.3390/plants12061384

**Published:** 2023-03-20

**Authors:** Wei Zhu, Zhengqing Xie, Zhenni Chu, Yakun Ding, Gongyao Shi, Weiwei Chen, Xiaochun Wei, Yuxiang Yuan, Fang Wei, Baoming Tian

**Affiliations:** 1 Henan International Joint Laboratory of Crop Gene Resource and Improvements, School of Agricultural Sciences, Zhengzhou University, Zhengzhou 450001, China; 2 Institute of Horticulture, Henan Academy of Agricultural Sciences, Graduate T&R Base of Zhengzhou University, Zhengzhou 450002, China

**Keywords:** apical dominance, auxin, *Brassica rapa*, chromatin remodeling factor, cytokinin, DNA methylation

## Abstract

The SHPRH (SNF2, histone linker, PHD, RING, helicase) subfamily belonging to ATP-dependent chromatin remodeling factor is the effective tumor-suppressor, which can polyubiquitinate PCNA (proliferating cell nuclear antigen) and participate in post-replication repair in human. However, little is known about the functions of SHPRH proteins in plants. In this study, we identified a novel SHPRH member *BrCHR39* and obtained *BrCHR39*-silenced transgenic *Brassica rapa*. In contrast to wild-type plants, transgenic *Brassica* plants exhibited a released apical dominance phenotype with semi-dwarfism and multiple lateral branches. Furthermore, a global alteration of DNA methylation in the main stem and bud appeared after silencing of *BrCHR39*. Based on the GO (gene ontology) functional annotation and KEGG (Kyoto encyclopedia of genes and genomes) pathway analysis, the plant hormone signal transduction pathway was clearly enriched. In particular, we found a significant increase in the methylation level of auxin-related genes in the stem, whereas auxin- and cytokinin-related genes were hypomethylated in the bud of transgenic plants. In addition, further qRT-PCR (quantitative real-time PCR) analysis revealed that DNA methylation level always had an opposite trend with gene expression level. Considered together, our findings indicated that suppression of *BrCHR39* expression triggered the methylation divergence of hormone-related genes and subsequently affected transcription levels to regulate the apical dominance in *Brassica rapa*.

## 1. Introduction

Chromatin dynamics play substantial roles in regulating the transcription pattern and controlling gene expression [1]. Structurally, chromatin is a highly dynamic DNA-protein complex, which is necessary for genome replication, transcription and epigenetic states, and DNA repair in plants [2,3]. The chromatin remodeling process affects the DNA replication, recombination, and other aspects through changing the position and controlling the structure of nucleosomes, and finally perceives the environmental stimuli to strictly regulate the growth and development [2,4,5]. Plants have evolved large amounts of unique chromatin remodeling complexes to enable epigenetic mechanism in this way, which liberates the tightly wrapped DNA and arises the transcriptional initiation [6,7]. Prominent chromatin remodelers are ATP-dependent, of which the SWI/SNF (switching defective/sucrose non-fermenting) family was the first and most studied chromatin remodeling complex [8]. Furthermore, the SNF2 (sucrose non-fermenting2) family was a crucial chromatin remodeling factor, which serves as the core catalytic subunit of chromatin remodeling complex to alter the chromatin accessibility, subsequently affecting gene expression to participate in plant development [9,10].

SNF2 proteins usually utilize the energy from ATP hydrolysis for nucleosome replacement and restructure to disturb the relationship between DNA and histone octamers [11,12,13]. SNF2 family members shared a common conserved domain, which possesses ATPase activities, ensuring the normal remodeling reactions [14]. In addition to the conserved ATPase domain, the amino terminal and carboxyl terminal of SNF2 proteins exist in different domains that trigger the specific protein interactions and developmental regulations. These domains can further divide the SNF2 family into six groups and eighteen subfamilies in *Arabidopsis thaliana* genome, such as the INO80 (inositol requiring 80), the Lsh (lymphoid-specific helicase), and the SHPRH subfamilies [15]. At present, the functional studies of chromatin remodeling factors are mainly focused on mammal animals and microorganisms, but little is known in plants.

Chromatin remodeling and DNA methylation are inextricably linked, which belong to important epigenetic processes in plants. Chromatin remodeling could be largely influenced by DNA methylation through promoting transcription activation to methylated cytosine [16]. DNA methylation is a critically important genetic mechanism without changing the DNA sequences, in which the methyl group can covalently bind to the cytosine of genome DNA, conferring the abilities of regulating plant growth and development [17,18]. Plant DNA methylation is defined in CG, CHG, and CHH types (H represents A, C or T) according to the sequence context. In general, different DMTases (DNA methyltransferases) undertake DNA methylation in different sequence contexts. For example, the maintenances of CG and CHG methylation are required for MET1 (DNA methyltransferase1) and CMT3 (chromomethylase3), respectively [19]. The CMT2, DRM1, and DRM2 (domain rearranged methyltransferase1 and 2) are necessary for CHH methylation [19,20]. Basically, DNA methylation is negatively correlated with transcription. Compared with conventional chromatin, the methylated DNA represses gene expression more effectively due to the assembly of their special nucleosome structures [21]. In particular, DNA methylation in promoter and enhancer regions can also inhibit gene expression by changing chromatin structure and disrupting transcription initiation [22].

Recent studies have demonstrated that at least four subfamilies of SNF2 family participate in the regulation of DNA methylation, although their molecular mechanisms are largely unknown [23]. Lsh protein, a member of the SNF2 family, assists DNMT1 in recruiting replication forks and initiating the de novo methylation via interacting with UHRF1 (ubiquitin-like containing PHD and ring finger domain 1) [24]. The other SNF2 protein, DDM1 (decreased in DNA methylation1), is essential for the DNA methylation process and is necessary for the formation of CHH methylation [25]. Furthermore, *OsDDM1*-defecient resulted in the decrease in histone H3K9me2 and increase in the heterochromatic small RNA level, implicating that *OsDDM1* antagonizes RdDM (RNA-directed DNA methylation) pathway at the heterochromatin level in rice [26]. The DRD1 (defective in RdDM1) subfamily members CLSYs (CLASSYs) can function as locus-specific regulatory factors in RdDM pathway to facilitate epigenetic diversity in plant development. Briefly, CLSY1 and CLSY2 are the prerequisites for the connection of SHH1 (Sawadee homeodomain homolog1) with Pol IV (RNA polymerase IV) complex. In addition, coupling of Pol IV targeted by these CLSYs relies on H3K9me2; nevertheless, CG methylation is required for CLSY3 and CLSY4 [27].

*BrCHR39* belongs to the SHPRH subfamily. *BrCHR39* is homologous with *AtCHR39* (AT3G54460) in *Arabidopsis thaliana*, which is the closest ortholog to the human *SHPRH* gene. SHPRH is identified as an effective DNA damage repair protein, which can activate the template switching pathway through accelerating PCNA polyubiquitination [28,29]. Additionally, SHPRH can act as DNA translocase to refrain DNA replication under replication stress [30,31]. To date, several studies have uncovered that *SHPRH* functions in an epigenetic-dependent manner. SHPRH was recruited to the nucleolar rDNA (ribosomal DNA) promoter due to the PHD (plant homeodomain) to recognize the epigenetic markers for enhancing rRNA transcription in mammals [32,33]. Nevertheless, how the SHPRH subfamily functions through affecting epigenetic processes in plants remains largely unknown.

In this study, we cloned a novel SHPRH member identified as *BrCHR39* and obtained transgenic lines by RNAi (RNA interference) technique in *Brassica rapa.* Then, the WGBS (whole-genome bisulfite sequencing) and qRT-PCR analyses were performed between wild-type plants and RNAi-*BrCHR39* plants. The transgenic lines exhibited a semi-dwarfism phenotype with multi-branches. Methylome analyses supported the fact that differentially methylated auxin and cytokinin genes were responsible for releasing apical dominance. In addition, *BrCHR39*-induced expression divergence had occurred, in which hormone-related genes were downregulated in stems and upregulated in lateral buds. These results suggested that chromatin remodeling factor *BrCHR39* regulated the methylation status of auxin- and cytokinin-related genes to modulate apical dominance, providing new insights into how the SHPRH subfamily affects DNA methylation to participate in the regulation of plant growth and development.

## 2. Results

### 2.1. BrCHR39 Confers the Maintenance of Apical Dominance in Brassica rapa

To investigate the function of *BrCHR39* in *B. rapa* plants, we constructed a RNAi-*BrCHR39* recombinant vector and obtained T_1_ transgenic lines (Figure S1a,b). Then, T_2_ transgenic plants were screened and identified at the transcription and DNA levels. The relative expression of *BrCHR39* gene was significantly decreased in transgenic plants compared with wild-type plants (Figure S1c,d), and from this, we selected three T_2_ lines, Ri-3, Ri-4, and Ri-9, for further analysis.

**Figure 1:**
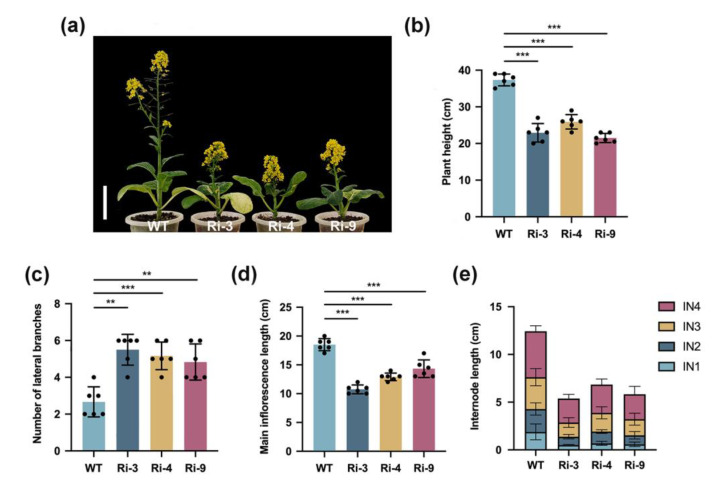
Phenotypic analysis of wild-type and *BrCHR39*-silenced plants. (**a**) Comparison of the phenotypes between 8-week-old wild-type and T_2_ transgenic plants. Scale bar = 10 cm. (**b**–**e**) Comparison of plant architecture-related traits, including plant height (**b**), number of lateral buds (**c**), main inflorescence length (**d**), and internode (IN) length (**e**) between 8-week-old wild-type and T_2_ transgenic plants. All values are presented as the means of six replicates ± standard deviation (SD). Significant differences are determined by Student’s *t*-test (** *p* < 0.01; *** *p* < 0.001).

After 8 weeks of growth, RNAi-*BrCHR39* plants showed a phenotype of weakened apical dominance with semi-dwarfism and excess of lateral branches in contrast to wild-type plants (Figure 1a). Dwarfism due to reduced *BrCHR39* expression was attributed to the shortening of the main inflorescence length and basal stem (Figure 1b,d,e). Furthermore, the average numbers of lateral branches in transgenic plants and wild-type plants were about 2.6 and 5.2, respectively, suggesting a two-fold greater change (Figure 1c). These results indicated that *BrCHR39* positively regulates the apical dominance in *Brassica rapa*.

### 2.2. Profiles of Genome-Wide DNA Methylation in RNAi-BrCHR39 Plants and Wild-Type Plants

Chromatin remodeling factors are generally associated with activation and inhibition of transcription, DNA methylation, and cell cycle [34]. For example, chromatin remodeling factor *PKL* (*PICKLE*) had a significant effect on DNA methylation at more than half of the RdDM loci [23]. To capture the DNA methylation dynamics after silencing *BrCHR39*, we performed WGBS in the main stems and buds from transgenic plants and wild-type plants (hereafter referred to as s_stem, wt_stem, s_bud, and wt_bud). In total, WGBS generated clean reads that ranged from 59,420,634 to 67,853,544 after the pre-process (Table 1). Then, the clean reads were mapped to the *B. rapa* genome with a range of 47.74–48.45% (Table 1). Importantly, the bisulfite conversion rates were over 99.7% in all samples, suggesting that the methylome data were reliable and accurate.

**Figure 2:**
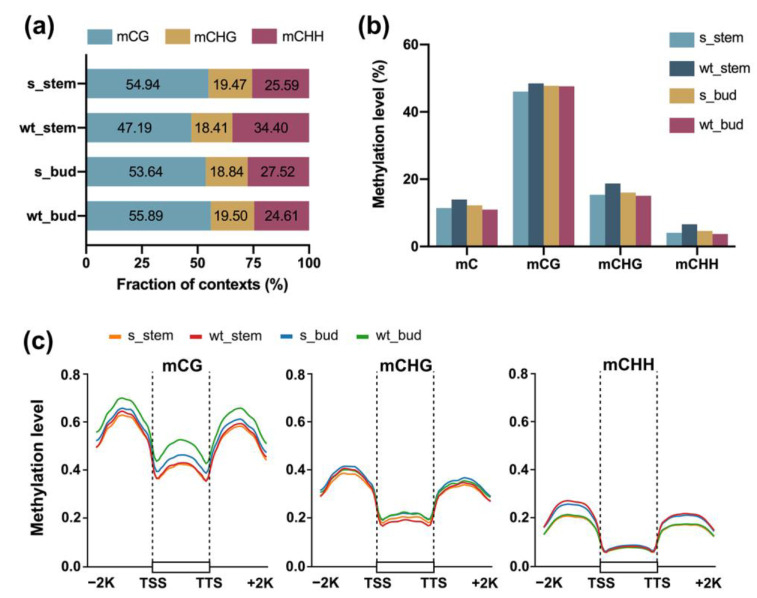
DNA methylation landscape of wild-type and *BrCHR39*-silenced plants. (**a**) Proportion of mCG, mCHG, and mCHH contexts in the stem and bud of wild-type and transgenic plants. (**b**) The DNA methylation levels of mCG, mCHG, and mCHH contexts of the stem and bud in wild-type and transgenic plans. (**c**) Distribution of DNA methylation levels for all samples in different genomic regions. TSS, transcription start site; TTS, transcription termination site.

Furthermore, DNA methylation was identified in three cytosine sequence contexts and harbored the highest level in CG context for all samples (Figure 2a). Additionally, average genome-wide methylation levels of CG, CHG, and CHH were 47.5%, 16.3%, and 4.8%, respectively (Figure 2b). Of these, the methylation level of CG context was the highest, which was consistent with methylation proportion. Moreover, we compared the changes in methylation level in the gene body and flanking region to explore the relationship between DNA methylation and gene expression. As shown in Figure 2c, the methylation levels in three sequence contexts sharply dropped around the TSS (transcription start site) and the TTS (transcription termination site), showing higher levels in the 5′ and 3′ flanking regions, but lower levels in the gene body regions. The genic regions with active transcription were accompanied with lower methylation level, suggesting that DNA methylation may have an inhibitory effect on gene expression (Figure 2c). Significantly, CG context exhibited the highest methylation level across gene body and flanking region, implying that the increase in CG methylation contributed most to the increase in DNA methylation, and even the change in gene expression.

### 2.3. Hypermethylated Auxin-Related DMGs Resulted in Semi-Dwarfism in RNAi-BrCHR39 Plants

**Figure 3:**
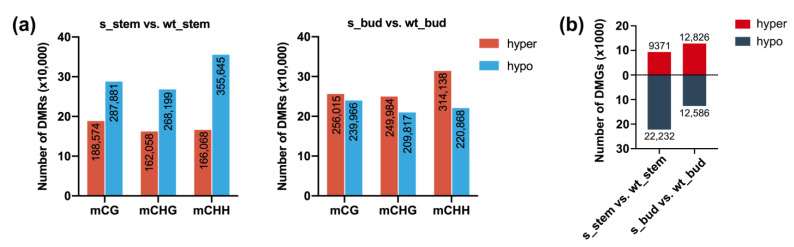
DMRs (differentially methylated regions) analysis of the stem and bud from wild-type and *BrCHR39*-silenced plants. (**a**) The number of DMRs in s_stem vs. wt_stem and s_bud vs. wt_bud. (**b**) The number of DMGs (DMR-related genes) in s_stem vs. wt_stem and s_bud vs. wt_bud. Hyper, hypermethylated. Hypo, hypomethylated.

To further explore the regulatory network between *BrCHR39* and apical dominance, we analyzed the DMRs (differentially methylated regions) in the main stem and bud of wild-type and transgenic *B. rapa*. A total of 516,700 hyper-DMRs and 911,725 hypo-DMRs were identified in s_stem vs. wt_stem (Figure 3a). In comparison to the wild-type plants, we identified 820,137 hyper-DMRs and 670,651 hypo-DMRs in the bud from transgenic plants (Figure 3a). All DMRs were classified into three contexts, and the CHH context contributed the greatest DMR number (Figure 3a). Furthermore, we selected genes with functional overlap of at least 1 bp with DMRs and defined as DMGs (DMR-related genes). Among the 9371 hyper-DMGs and 22,232 hypo-DMGs identified in the s_stem vs. wt_stem, 12,826 became hyper-DMGs and 12,586 became hypo-DMGs in s_bud vs. wt_bud (Figure 3b).

**Figure 4:**
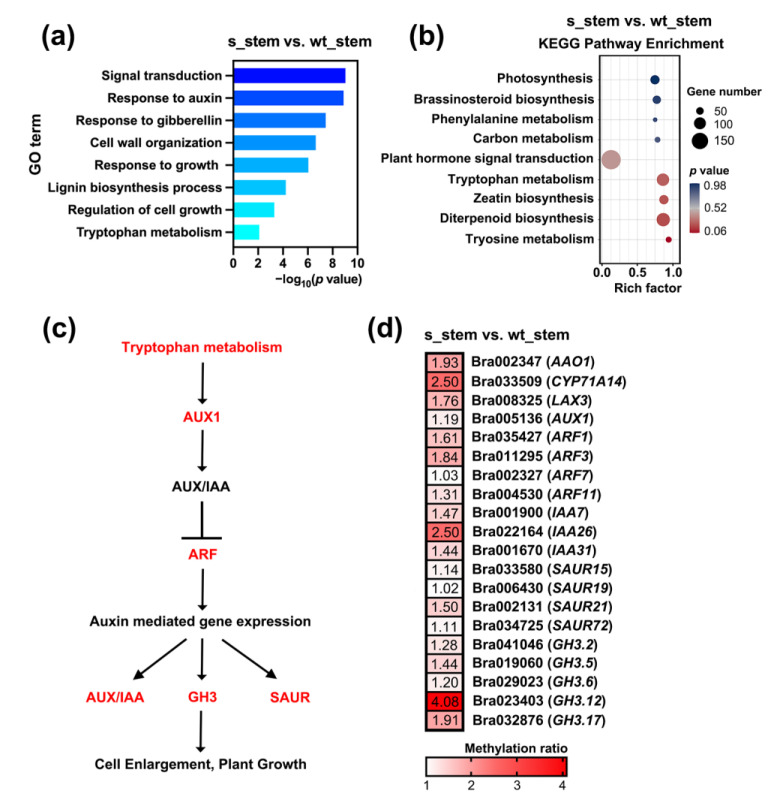
Functional analysis of DMGs in s_stem vs. wt_stem. (**a**) GO (gene ontology) functional enrichment of the DMGs in s_stem vs. wt_stem. (**b**) KEGG (Kyoto encyclopedia of genes and genomes) pathway analysis of the DMGs in s_stem vs. wt_stem. (**c**) The schematic diagram of auxin signal transduction pathway. (**d**) The methylation levels of auxin-related DMGs in s_stem vs. wt_stem. The hypermethylated DMGs identified in this study are marked in red. Arrows and “T” bars represent positive and negative regulation, respectively.

To understand the potential regulation in plant height, GO analysis was conducted to identify the enriched biological processes in response to the silencing of *BrCHR39* gene. Apparently, a significant amount of DMGs in s_stem vs. wt_stem was dramatically enriched in GO terms (*p* < 0.01) associated with plant hormone and growth regulation, such as response to auxin (GO:0009733), response to gibberellin (GO:0009739), response to growth (GO:0007165), and regulation of cell growth (GO:0001558), suggesting a greater response of hormonal changes in transgenic plants (Figure 4a, Table S2). Accordingly, further KEGG analysis showed that hormone-related pathways were significantly enriched (Figure 4b, Table S3).

Among the plant hormone signal transduction pathway, the largest number of DMGs was in auxin signaling pathway. High content of auxin can promote plant growth and maintain apical dominance through enhancing plant height directly [35,36]. As expected, we observed that most of DMGs in auxin-related pathway were hypermethylated (Figure 4c). In detail, DMGs involved in tryptophan metabolism pathway, such as *AAO1* (*Arabidopsis aldehyde oxidase1*) and *CYP71A14* (*Cytochrome P450 71A14*), which participated in the auxin biosynthesis process had higher methylation levels in transgenic plants compared to wild-type plants (Figure 4c). In addition, some important DMGs in auxin signal transduction pathway were differentially hypermethylated in s_stem vs. wt_stem (Figure 4c). For example, the methylation levels of auxin responsive gene families including *IAA* (*Indole-3-acetic acid*), *SAUR* (*Small auxin upregulated RNA*), and *GH3* (*Gretchen Hagen3*) genes notably increased in transgenic plants (Figure 4c). These results supported the notion that the inhibition of stem growth under silencing of *BrCHR39* gene was mainly due to the hypermethylation of auxin-related DMGs.

### 2.4. Hypomethylated Auxin and Cytokinin DMGs Promoted Bud Outgrowth in RNAi-BrCHR39 Plants

**Figure 5:**
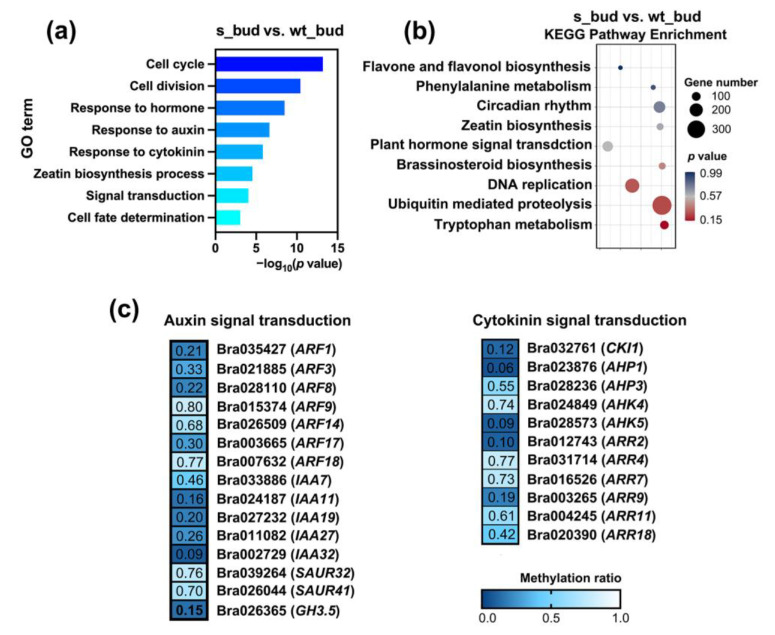
Functional analysis of DMGs in s_bud vs. wt_bud. (**a**) GO functional enrichment of the DMGs in s_bud vs. wt_bud. (**b**) KEGG pathway analysis of the DMGs in s_bud vs. wt_bud. (**c**) The methylation levels of auxin- and cytokinin-related DMGs in s_bud vs. wt_bud.

Previous studies have evidenced that the excess of buds had been attributed to the suppression of growing apical dominance [37]. Auxin and cytokinin were the primary factors in the regulation of local bud outgrowth through an independent manner or reciprocal regulation [38]. To reveal the methylation changes in axillary buds before and after silencing of *BrCHR39*, we performed GO functional analysis in s_bud vs. wt_bud. As shown in Figure 5a and Table S4, significantly enriched GO terms, such as cell cycle (GO:0007049), cell division (GO:0051301), response to auxin (GO:0009733), and response to cytokinin (GO:0009735) were found in s_bud vs. wt_bud, indicating that cell growth and phytohormones controlled bud outgrowth by regulating the methylation level of the DMGs. In accordance with this, hormone-related pathways were also enriched in KEGG analysis (Figure 5b, Table S5). Given that the plant hormone played an indispensable role in the stem growth, we emphatically analyzed the DMGs in plant hormone signal transduction pathway. In contrast to stem, auxin- and cytokinin-related genes contributed to the highest proportion in the hormone signaling pathway of s_bud vs. wt_bud. In contrast to wild-type plants, decreasing methylation levels of genes in auxin signal transduction pathway occurred in transgenic plants (Figure 5c). Additionally, the methylation levels of the components in cytokinin signal transduction pathway including histidine kinase *CKI1*, histidine phosphotransfer *AHPs*, and two-component response regulator *ARRs* tended to decrease in transgenic plants (Figure 5c). Collectively, these results implied that auxin and cytokinin DMGs were heavily hypomethylated after silencing of *BrCHR39* gene, contributing to the increased lateral bud number.

### 2.5. Expression Profiles of DMGs

**Figure 6:**
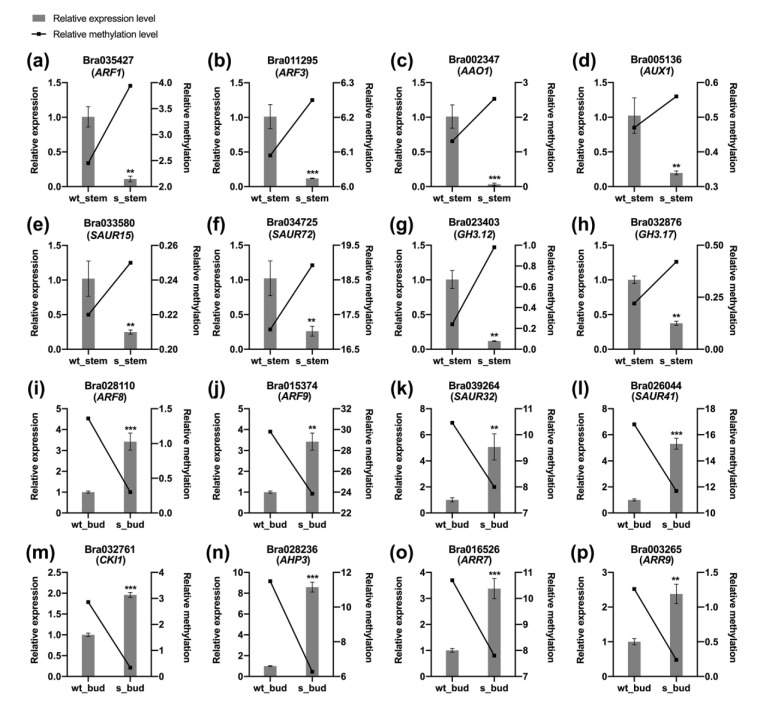
Relationship between the expression level and methylation level of DMGs. (**a**–**h**) The qRT-PCR (quantitative real-time PCR) analysis of eight auxin-related DMGs in the stems from wild-type and transgenic plants. (**i**–**p**) The qRT-PCR analysis of eight auxin- and cytokinin-related DMGs in the buds from wild-type and transgenic plants. The 2^−ΔΔCt^ method was used to calculate the relative expression levels of genes. Error bars indicate the means of three replicates ± SD. Significant differences are determined by Student’s *t*-test (** *p* < 0.01; *** *p* < 0.001). Relative expression was set on the left Y-axis with grey columns and relative methylation was on the right Y-axis with black polyline.

The processes of genic transcription and DNA methylation are closely interwoven [21]. Hypermethylation is usually accompanied with lower gene expression level, whereas hypomethylation results in the occurrence of higher expression level [39]. To further understand the relationship between the methylation level and transcription level of DMGs, qRT-PCR was applied to examine the gene expression level. We randomly selected sixteen DMGs for the qRT-PCR analysis, of which eight auxin-related DMGs were detected in the main stem, and eight auxin- or cytokinin-related DMGs were detected in the lateral bud. Consistent with previous studies, hypermethylated auxin DMGs showed negative expression patterns in the stem of transgenic plants (Figure 6a–h). For example, *AAO1* gene involved in auxin biosynthesis process, auxin influx transmembrane transporter *AUX1*, auxin response factors *ARF1* and *ARF3*, and auxin responsive genes *SAUR15*, *SAUR72*, *GH3.12*, and *GH3.17* were downregulated in the stem after suppressing the *BrCHR39* expression (Figure 6a–h). Moreover, compared with wild-type plants, genes involved in auxin signaling and cytokinin signaling were highly expressed with decreased methylation levels in the bud of transgenic plants (Figure 6i–p). In conclusion, these data suggested that the differential DNA methylation of auxin- and cytokinin-related genes resulted in the weakened apical dominance.

## 3. Discussion

Chromatin remodeling factors play indispensable roles through unwinding the compacted chromatin state in plants. The SNF2-type ATPases are thoroughly studied chromatin remodelers that have specific functions according to their unique domains. For example, the *pkl* mutant had lower plant height and retarded root meristem [40,41]. Loss function of *AtINO80* led to the smaller organ and later flowering than wild-type *Arabidopsis* [42]. However, the functions of SHPRH members in plants remain unclear.

Accumulating evidences showed that HLTF (helicase-like transcription factor) and SHPRH, the representative members of SHPRH subfamily, are effective tumor suppressors with non-reductant functions [43,44]. Both have been demonstrated to improve the sensitivity of DNA damage and rearrange the large chromosomes [45]. To further understand the roles of SHPRH proteins in plants, we silenced *BrCHR39* gene and generated transgenic *B. rapa* lines via RNAi approach. Suppressing of *BrCHR39* expression resulted in pleiotropic phenotype including weakened apical dominance, semi-dwarfism, and excessive branches (Figure 1). Furthermore, the main inflorescence length and representative basal internode length were significantly shortened in transgenic plants compared with wild-type plants, suggesting that the holistically lower plant height was attributed to the main inflorescence and internode (Figure 1d,e). Conversely, the lateral bud exhibited strengthen growth, and finally induced multiple branches in *BrCHR39*-silenced plants (Figure 1c). These results help in preliminarily characterizing the function of *BrCHR39* gene, which positively regulates the apical dominance.

SHPRH subfamily has been found to be actively involved in epigenetic processes in mammals. Human HLTF can alter its expression in various cancers through the hypermethylation in the promoter region [46]. Given that several chromatin remodeling factors and DNA methylation are interlinked and synergistically regulate many developmental processes during plant growth, we speculate that SHPRH has an underappreciated effect on DNA methylation. The present study identified genome-wide methylation levels in the main stems and buds between the wild-type and transgenic plants by performing WGBS. The average bisulfite non-conversion rates of all the samples were 0.27%, which were acceptable and consistent with previous studies [47]. Regardless of whether silencing of *BrCHR39* occurred, CG methylation always occupied the highest level in the main stems and buds (Figure 2a). Moreover, CG methylation level maintained the highest level in each sample, indicating that CG context played a dominant role in the methylation alterations (Figure 2b,c). Consistent with these data, several studies have shown that the highest methylation density occurred in CG context in rapeseed plants [48,49,50]. DNA methylation in the CG and CHG contexts is the most common phenomenon in land plants, although DNA methylation patterns differ among species. In particular, CG methylation contributes to more than 50% of the total cytosine methylation in angiosperms [51]. The CG methylation is crucial for gene silencing and is progressively imprinted via mitotic and meiotic cell divisions [52]. In addition, we specially observed that DNA methylation displayed the lowest level in the intergenic region, implying an opposite pattern between DNA methylation and transcription (Figure 2c).

To further dissect the potential regulatory mechanism of *BrCHR39* in regulating apical dominance, GO and KEGG functional analyses were applied to enrich the underlying pathways. A total of 9371 hypermethylated DMGs and 22,232 hypomethylated DMGs were identified in s_stem vs. wt_stem (Figure 3b). The plant hormone signal-related pathways were specifically enriched, namely, the DNA methylation states of hormone-related genes were remarkably changed after silencing of *BrCHR39* gene (Figure 4a,b). Further inspections showed that the auxin-related DMGs accounted for the majority of plant hormone signal transduction pathway. Therefore, we narrowed our methylome analysis to those auxin signal transduction pathway-related genes. Of these DMGs, most hypermethylated to varying degrees, suggesting that silencing of *BrCHR39*-induced hypermethylated auxin genes helps in releasing apical dominance (Figure 4c,d). Although gibberellin- and brassinosteroid-related genes were also enriched in the plant hormone signal transduction pathway, the methylation status of most genes showed no significant difference before and after silencing of *BrCHR39*, indicating that both rarely contributed to the regulation of apical dominance. The apical dominance is established by stem apical meristem, which is the primary plant shoot and exerts a regulatory role to arrest axillary buds [35,53,54]. Plant hormone is well known to shape apical dominance, which mediates signal transduction across the internal plant, and thereby manipulates the growth cycle and final volume [55]. Auxin is the primary regulator in the formation of apical dominance, which is synthesized in shoot apex and flows downward to boost stem growth, indirectly impairing lateral bud outgrowth [56,57]. Therefore, we consider that suppressing of *BrCHR39* expression reduced the plant height mainly due to the dynamic changes in auxin.

Apical dominance is a phenomenon in which the preferred growth of intact main shoot apex is accompanied with inhibited axillary bud outgrowth. To explain the occurrence of excessive branches in transgenic plants, we performed functional analyses in s_bud vs. wt_bud. The numbers of DMRs and DMGs were first counted in Figure 3a,b. Consistent with GO and KEGG analyses in s_stem vs. wt_stem, plant hormone signal transduction pathway was also enriched in s_bud vs. wt_bud, suggesting that the hormone governed predominantly in the regulation of bud outgrowth (Figure 5a,b). Intriguingly, DMGs associated with auxin and cytokinin signal transduction pathway were significantly enriched with lower DNA methylation level, which was a pronounced difference with results in s_stem vs. wt_stem (Figure 5c). A potential explanation for this discrepancy was the opposite growth trend of stem and bud in transgenic plants, which perhaps caused differences in DNA methylation levels. Additionally, functional enrichment showed that the buds were specifically enriched with cytokinin-related DMGs in transgenic plants, which likely linked with the substantial role of cytokinin in regulating branching. It is generally acknowledged that local cytokinin acts in conjunction with auxin to promote cell division and differentiation in axillary meristems [58,59]. Furthermore, the accumulation of cytokinin can allow buds to escape apical dominance for normal growth [60,61]. Considered together, these data indicated that hypomethylated auxin and cytokinin DMGs synergistically enhanced the ability of bud outgrowth.

Transcriptional differences were often accompanied with changes in methylation status, whether in plants or animals [62,63]. Plants ubiquitously modulate their physiological processes and developmental mechanisms through changes in transcription to trigger responses at an epigenetic level, such as in DNA methylation [64,65]. Clearly, the hypermethylation is generally supposed to impair the gene expression and vice versa [21,66]. Our results suggested that the DNA methylation levels of DMGs negatively correlated with the expression levels, regardless of the stem or bud, which is in agreement with the previous studies (Figure 6). In detail, considering the general function of DNA methylation in inhibiting transcription, hypermethylated auxin DMGs were supposed to negatively express in the stem and subsequently result in lower plant height of transgenic plants. The hypomethylated auxin and cytokinin DMGs with active gene expression led to a bushier phenotype.

Therefore, our findings indicated that hormone-related DMGs, particularly auxin DMGs, were regulated by reshaping the DNA methylation landscape to modulate apical dominance after suppressing the *BrCHR39* expression.

## 4. Materials and Methods

### 4.1. Plant Materials and Growth Conditions

The selfing *B. rapa* DH line (cxl-45-05) was used in this study. Seeds were soaked for germination and grown in pots filled with a mixture of soil:vermiculite (3:1, *v*/*v*) in a growth chamber with 21/18 °C (day/night), 16/8 h (light/dark), and 200 μmol/m^2^/s light density.

### 4.2. Generation of Transgenic Plants

The sequence of the *BrCHR39* (Bra014815) gene was provided by the BRAD database (http://brassicadb.cn/, accessed on 17 October 2021). A 196 bp fragment of the CDS (coding sequence) in *BrCHR39* was amplified and ligated into the pHELLSGATE 12 vector. The recombinant vector was transformed into *B. rapa* plants using the *A. tumefaciens*-mediated floral dip method [67]. The seeds collected from the dipped plants were screened on half-strength MS (Murashige and Skoog) media containing 100 mg/L kanamycin. Three T_2_ homozygous lines (Ri-3, Ri-4, Ri-9) were used for further study. Details of all the primers used are provided in Table S1.

### 4.3. Whole-Genome Bisulfite Sequencing (WGBS) and Data Analysis

The main stems and buds of 8-week-old *BrCHR39*-silenced plants and wild-type plants were collected. Total DNA was extracted using a plant genomic DNA isolation mini kit (Vazyme, Nanjing, China) according to the manufacturer’s procedure. The DNA purification was measured by the A260/280 ratio using a spectrophotometer (IMPLEN, Westlake Village, CA, USA). The DNA samples were fragmented using sonication, and then subjected to end repair and adenylation. These DNA fragments were subsequently treated two times with bisulfite. Furthermore, the obtained single-strand DNA fragments were amplified using KAPA HiFi HotStart Uracil + ReadyMix (2X). Library concentration was quantified, and the insert size was checked. The clustering of the index-coded samples was performed on a cBot Cluster Generation System using Truseq PE Cluster Kit v3-cBot-HS (Illumina, San Diego, CA, USA) following the manufacturer’s instructions. Paired-end 2 × 150 bp sequencing was performed on an Illumina HiSeq 4000 platform at LC Life Sciences Ltd. (Beijing, China). To pre-process the raw data, cutadapt and in-house Perl scripts were used to remove the reads with contaminated adapters, low-quality reads, and undetermined reads. Subsequently, the sequence quality was tested by FastQC (http://www.bioinformatics.babraham.ac.uk/projects/fastqc/, accessed on 20 August 2022). The reads that passed quality control were mapped to the *B. rapa* genome (v3.1) using Bismark (version 0.15.1) software with default parameters. Finally, the reads were further deduplicated using SAMtools (http://samtools.sourceforge.net/, accessed on 1 September 2022).

The methylation level was identified via a sliding-window approach, which was determined by the ratio of the number of reads supporting mC (methylated cytosines) over mC and non-mC (total cytosines) using in-house Perl scripts and MethPipe [68].

### 4.4. Differentially Methylated Region Analysis

The DMRs were identified using the methylKit R package, which is theoretically similar to the above approach for detecting the methylation level with a read coverage ≥ 5, an absolute difference in methylation level ≥0.1 or FC (fold change) ≥2, and FDR (false discovery rate) <0.05. The window was set to 1000 bp and the step length was 100 bp. Then, the probabilities were measured using the Fisher test. Additionally, the different types of DMRs (mCG, mCHG, and mCHH) were identified following a published method [69]. The DMGs were classified as genes having at least 1 bp of functional overlap with significant DMRs.

### 4.5. GO and KEGG Enrichment Analysis of DMR-Related Genes

All the DEGs were subjected to gene ontology functional analysis (http://www.geneontology.org/, accessed on 28 November 2022) and Kyoto encyclopedia of genes and genomes pathway enrichment analysis (http://www.genome.jp/kegg/, accessed on 28 November 2022). The results of the GO functional analysis and KEGG pathway enrichment analysis were considered significant with *p*-value < 0.05.

### 4.6. Quantitative Real-Time PCR

Total RNA of the main stems and buds from 8-week-old *BrCHR39*-silenced plants and wild-type plants was extracted using the TRIzol reagent (Invitrogen, Carlsbad, CA, USA), and the first-strand cDNA (complementary DNA) was synthesized using the HiScript^®^ Ⅲ RT SuperMix for qPCR Kit (Vazyme, Nanjing, China). The quantitative real-time PCR was then performed on a LightCycler480 machine (Roche, Basel, BS, Switzerland) using the SYBR Green Master Mix (Vazyme, Nanjing, China). The relative expression levels of genes were determined using the 2^−∆∆CT^ method [70], and the values were shown as the means of three biological replicates *±*SD (standard deviation). The *actin7* gene (Bra009081) was used as an internal control. All the primers used for qPCR are listed in Table S1.

## 5. Conclusions

Based on the above observations, chromatin remodeling factor *BrCHR39* functioned as the positive regulator in the formation of apical dominance. Further global bisulfite sequencing of stems and buds from wild-type and RNAi-*BrCHR39* showed that auxin-related DMGs were hypermethylated with lower gene expression in the stem, and the DMGs in auxin or cytokinin signal transduction pathway were hypomethylated with higher expression level in the bud after suppressing the *BrCHR39* expression. Therefore, we speculated that the methylation status of auxin- and cytokinin-related genes was changed to execute central roles in the regulation of apical dominance after the perception of decreased *BrCHR39* expression. Our findings provide great evidence for the communication between SHPRH subfamily and DNA methylation.

## Figures and Tables

**Table 1:** Summary of whole-genome bisulfite sequencing data.

Samples	Raw Reads	Clean Reads	Mapped Reads	Mapping Rate (%)	Bisulfite Conversion Rate (%)
s_stem	67,906,410	67,853,544	32,396,178	47.74	99.75
s_stem	67,906,410	67,853,544	32,396,178	47.74	99.75
wt_stem	63,995,464	63,939,594	30,980,118	48.45	99.71
s_bud	67,422,054	67,365,438	32,592,740	48.38	99.73
wt_bud	59,469,540	59,420,634	28,819,630	48.50	99.72

## Data Availability

The WGBS data for this study were deposited in the NCBI Sequence Read Archive (SRA) under the Bioproject accession number PRJNA811755.

## Supplementary Materials

The following supporting information can be downloaded at: https://www.mdpi.com/article/10.3390/plants12061384/s1. Figure S1: Screening and identification of the T_1_ and T_2_ transgenic lines; Table S1: List of primers used in this study; Table S2: GO functional enrichment of the DMGs in s_stem vs. wt_stem; Table S3: KEGG pathway analysis of the DMGs in s_stem vs. wt_stem; Table S4: GO functional enrichment of the DMGs in s_bud vs.wt_bud; Table S5: KEGG pathway analysis of the DMGs in s_bud vs. wt_bud.
