# Supplementary material for: The Chromatin Remodeling Factor BrCHR39 Targets DNA Methylation to Positively Regulate Apical Dominance in Brassica rapa
Source: Plants (Basel). 2023 Mar 20;12(6):1384. doi: 10.3390/plants12061384 (PMC10051476; doi:10.3390/plants12061384)
Supplement: Supplementary file 1 [file plants-12-01384-s001.zip › Supplementary figure.pdf]

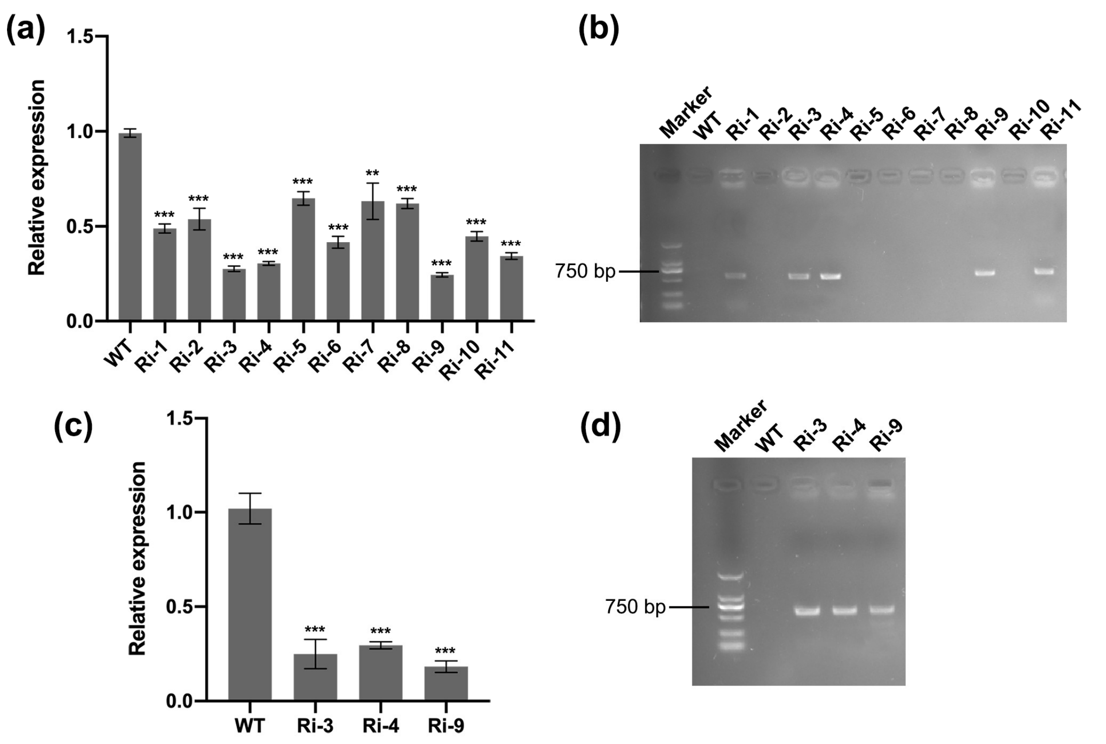

Figure S1. Screening and identification of the T_1 and T_2 transgenic lines.

(a) Relative expression of *BrCHR39* gene in T_1 transgenic plants. (b) Screening of T_1 transgenic plants at DNA level. Marker, 2000 bp. (c) Relative expression of *BrCHR39* gene in selected T_2 transgenic plants. (d) Screening of T_2 transgenic plants at DNA level. Marker, 2000 bp; All values are presented as the means of three replicates ± standard deviation (SD). Significant differences are determined by Student's *t*-test (**$p < 0.01$; ***$p < 0.001$).
